# Suffering and Salutogenesis: A Conceptual Analysis of Lessons for Psychiatry From Existential Positive Psychology (PP2.0) in the Setting of the COVID-19 Pandemic

**DOI:** 10.3389/fpsyg.2021.646334

**Published:** 2021-04-09

**Authors:** Ravi Philip Rajkumar

**Affiliations:** Department of Psychiatry, Jawaharlal Institute of Postgraduate Medical Education and Research, Pondicherry, India

**Keywords:** salutogenesis, positive psychology, COVID-19, mental health, psychiatry

## Abstract

The COVID-19 pandemic has had a widespread effect on the thoughts, emotions and behavior of millions of people all around the world. In this context, a large body of scientific literature examining the mental health impact of this global crisis has emerged. The majority of these studies have framed this impact in terms of pre-defined categories derived from psychiatric nosology, such as anxiety disorders, depression or post-traumatic stress disorder. These constructs often fail to capture the complexity of the actual experiences of the individuals being studied; more specifically, they describe these experiences exclusively in terms of disease, while neglecting their potentially adaptive or “salutogenic” aspects. Similarly, discussion of psychological assistance for these individuals has largely been confined to a reiteration of “evidence-based” psychological or pharmacological techniques which can be delivered using remote access technology. In the context of the COVID-19 pandemic, these approaches are likely to be of mixed efficacy. Conversely, “negative emotions” or distressing psychological experiences may actually be functional in the setting of a disaster or crisis, serving to minimize harm, maximize social coherence and compliance, and facilitate adherence to safety measures. The limitations of the “conventional” approach are, to a certain degree, inherent to the prevailing medical model of mental health. Beyond these considerations lies the concept of “salutogenesis,” a term which refers to the innate capacity of individuals to create and maintain health and well-being in the face of adversity. Using principles derived from the second wave of positive psychology (PP2.0), particularly its emphasis on the totality of human experience and the possibility of deriving meaning and character growth from suffering, this paper conceptually analyses the relevant aspects of salutogenesis and PP2.0, and proposes an alternate approach for addressing mental health concerns during the COVID-19 pandemic. Such an approach, while acknowledging the utility of the conventional medical-psychotherapeutic model in specific cases, reduces the risk of medicalizing human experience, and provides individuals and communities with opportunities for growth and adaptation. The benefits of this proposal could potentially extend far beyond the current crisis, offering an opportunity for the field of psychiatry and mental health research to move away from a purely “disease-centered” model.

## Introduction

The global pandemic of acute respiratory illness caused by the novel coronavirus SARS-CoV-2, officially designated COVID-19, has emerged as the single largest public health crisis of our times. In estimating the human cost of this pandemic, one must take into account not only the mortality and morbidity caused directly by the disease itself (Hozhabri et al., 2020), but its indirect adverse effects on the healthcare system (Dunham et al., 2020), and more importantly, the immense social and economic disruptions occasioned by quarantine, lockdown and “stay-at-home” orders (Schippers, 2020). Taken together, this has resulted in an exacerbation and accentuation of pre-existing social problems, including poverty, food insecurity, unemployment and violence against vulnerable populations such as women and children (Bong et al., 2020; Moen et al., 2020; Usher et al., 2020). These increases in adversity have been associated with the publication of several reports from all around the world, reporting increases in psychological distress both in the general population and in “high-risk” groups such as healthcare workers. Recent meta-analytic reviews have estimated that in the general population, around 22% of individuals experience significant symptoms of depression, 28% experience symptoms of anxiety, and 33% experience symptoms of post-traumatic stress disorder (PTSD) (Arora et al., 2020). The reported corresponding figures for healthcare workers are 22.8% for depressive symptoms and 23.2% for anxiety symptoms respectively (Pappa et al., 2020). Faced with these figures, several experts have described the current situation as a mental health crisis (The Lancet Public Health, 2020) or a threat to global mental health (Anjum et al., 2020), leading to a marked global increase in suicidal attempts and deaths (Dutheil et al., 2020). In turn, this has led to a wide variety of proposals on how to alleviate this crisis, and how best to provide mental health care to affected individuals (Muller et al., 2020; Soklaridis et al., 2020).

This rapid response to problems that could potentially affect millions of people is laudable. However, it obscures the fact that the medical or psychiatric paradigm may not be the most appropriate model when considering the complex phenomenon of psychological distress during a global pandemic. In this paper, the limitations of this paradigm, both in terms of equating psychological distress with mental disorder and in terms of the interventions offered, will first be outlined. Next, alternative conceptual approaches to the problem of psychological distress in the context of COVID-19 will be discussed. Following this, the key concept of salutogenesis and how it relates to these issues will be outlined, and the role of existential positive psychology–sometimes labeled “positive psychology 2.0” or “PP2.0”–in fostering salutogenesis, personal and community growth, and resilience in the face of this global health crisis will be described. A proposal for the implementation of strategies based on this approach will then be outlined, along with the potential benefits and risks of such strategies.

## The Rationale for an Alternative Approach to Psychological Distress in the Setting of the COVID-19 Pandemic

### Methodological Issues

Central to the approach advanced in this paper is the question of whether psychological distress, even when widespread, can be reduced to a “mental health crisis” or “mental health emergency.” The correct answer to this question requires an understanding of both the methodology adopted by researchers in this area, and the conceptual framework used to interpret the results obtained. From a methodological perspective, most–indeed, almost all–of the observational studies reporting “anxiety,” “depression,” or “PTSD” are based on the use of self-administered screening questionnaires. These instruments were originally designed for the rapid identification of people with a possible mental disorder in community or clinic samples, and consist of a limited number of items that are usually rated by the respondent using a Likert-type scale. Based on prior population-based research, a “cut-off point” is identified which separates individuals who are more or less likely to fulfill criteria for a particular psychiatric syndrome (Mughal et al., 2020). However–and this is the crucial point obscured by most literature on COVID-19 and mental health–the confirmation of such a syndromal diagnosis requires a second step, typically a structured interview by a trained clinician or researcher, using standard diagnostic criteria. Without this second step, any research relying on screening instruments alone is likely to overestimate the presence of a potential mental disorder. A global study of depression (Bromet et al., 2011) illustrates this point. In this study, individuals from 18 countries were first screened for possible depression and then subjected to a more in-depth, structured interview if they screened positive. It was found that in the ten developed countries, only 52% (14.6% of 28.1%) of those screening positive were confirmed to have syndromal depression; the corresponding figure for the eight developing countries was 56% (11.1% of 19.8%). In other words, only around 50–55% of individuals screening positive for “depression” fulfill the formal diagnostic criteria for major depressive disorder. Similar considerations apply to the use of screening instruments for anxiety disorders and PTSD. Furthermore, the choice of screening instrument itself can significantly influence how many individuals “screen positive” for a disorder; for example, hospital outpatients screened with the Hospital Anxiety and Depression Scale had a mean prevalence of depressive symptoms of 22%, while this rose to 36% if the Beck Depression Inventory was employed (Wang et al., 2017). From these considerations, it is clear that around half of “significant” symptoms of anxiety, depression or PTSD identified in observational studies of COVID-19 cannot be equated with a syndromal mental disorder. It is important to note the researchers conducting studies of mental health during the COVID-19 pandemic have acknowledged these limitations themselves, and that face-to-face interviews may not always be feasible in the context of social distancing or other safety measures (Lin et al., 2021). Therefore, the above considerations should not be taken as a dismissal of this valuable body of work. Rather, they should be understood as a cautionary note on the interpretation of the results thereby obtained, particularly by those not involved in the original research. It is clear that even individuals without a syndromal diagnosis experience significant psychological distress, but how can this distress be best understood?

### Conceptual Frameworks for Psychological Distress in the Context of a Pandemic, and Their Practical Implications

There are at least three explanatory models for psychological distress in the context of COVID-19 with some support from the scientific literature:

(A) Specific forms of psychological distress, particularly symptoms resembling those of depression (Anders et al., 2013) and obsessive-compulsive disorder (Rajkumar, 2020a), play a specific role in protecting individuals and communities from the threat of infectious diseases. In this model, these forms of psychological distress are part of the “behavioral immune system” that has evolved to protect the human race from the survival threat posed by pathogens (Shakhar, 2019).
(B) Psychological distress in these individuals is an understandable response to extreme degrees of social and economic adversity–in other words, “a normal response to an abnormal situation,” or an emotional and behavioral “cry for help” that arises when environmental threats overwhelm the individual’s limited resources and threaten his (or his family’s) survival or well-being. The function of these responses is to facilitate help-seeking, to minimize further harm, or to avoid danger. In these cases, though the experience of distress is aversive, it serves an adaptive function analogous to physical pain, unless a point is reached when help is not available or forthcoming–in which case a full-blown psychiatric syndrome results (Bateson et al., 2011; Hagen, 2011).
(C) Psychological distress in these individuals is part of a continuum between mental health (defined as the absence of significant psychiatric symptomatology) and syndromal mental disorders. Though individuals with these symptoms may not receive a formal diagnosis, they are suffering from a “sub-syndromal” or “sub-threshold” mental disorder in response to stress, which is associated with an increased subsequent risk of developing the corresponding syndromal disorder as well as increased disability and reduced quality of life (Diefenbach et al., 2003; Meeks et al., 2011). In current nosological systems, such individuals might receive a diagnosis of “adjustment disorder” (Strain, 2019).

For the sake of convenience, these may be designated the *evolutionary*, *social*, and *medical* models of psychological distress in response to a pandemic. It is important to note that these models are not mutually exclusive. Medical models of mental illness acknowledge the importance of social factors in influencing the onset and course of these disorders (Tibubos et al., 2019), and evolutionary models attempt to distinguish between adaptive and maladaptive responses to specific environmental circumstances (Taylor et al., 2011). Thus, from a theoretical perspective, all these models offer valuable insights and are not necessarily in conflict.

However, the choice of explanatory model is significant when it comes to deciding whether interventions are appropriate for these “symptoms,” and if so, what form these interventions should take. Most published literature to date has implicitly adopted the medical model, recommending the use of standard psychiatric treatments such as cognitive-behavioral therapy for the management of psychological distress (Qiu et al., 2020; Boldt et al., 2021). However, if certain forms of psychological distress are adaptive in nature, attempting to minimize them may do more harm than good. In the context of COVID-19, there is already evidence that a certain level of fear (“functional fear”) may positively influence public health compliance (Harper et al., 2020), while a lower-level of self-reported worry is associated with a lower likelihood of adherence to safety precautions (Barber and Kim, 2020). Labeling individuals with a “functional” level of fear as “symptomatic” and in need of treatment may thus, paradoxically, diminish their adherence to protective behaviors and lead to increased transmission of SARS-CoV-2. Similarly, if psychological distress is an understandable reaction to high levels of social hardship, then offering individual psychological interventions may delay, or even divert attention from, the provision of necessary social and economic support–which may include financial aid, unemployment benefits, or supports to individuals, families and communities with a prior socioeconomic vulnerability (Hagen, 2011; Banati et al., 2020; Rajkumar, 2020b). A further concern with “medical” modes of treatment, particularly if pharmacological treatment is offered (which is often the case where trained therapists are not available), is that some pharmacological agents have the undesirable adverse effect of blunting empathy, particularly empathy evoked by exposure to the suffering of others (Rutgen et al., 2019). This could lead to a reduced likelihood of cooperating with public health measures (Pfattheicher et al., 2020).

## Salutogenesis and Its Relevance to Psychological Distress

### The Concept of Salutogenesis

It is clear that there is more than one way to understand the distressing emotional responses experienced by individuals faced with the COVID-19 pandemic, and that an approach based on conventional psychiatric nosology may have significant limitations. However, it is possible to go even further by making use of the concept of salutogenesis–or, to be more accurate, the *salutogenic framework*. This notion, based on the work of the medical sociologist Aaron Antonovsky, sees health as existing on a continuum between disease and normality, also sometimes termed “ease” or “total health” (Lindstrom and Eriksson, 2005). From this perspective, the focus is not on the mechanism underlying sickness or disease, but on how one can best move from “disease” to “ease” (Antonovsky and Sagy, 2017).

The first tenet of this model is that *illness and entropy (referring to decline into ill-health)–are the rule rather than the exception*; to a certain extent, they are inevitable. Thus, it is not practical to attempt to address all known risk factors for a given disease, such as stressors–instead, the focus is on facilitating adaptation to the environment in a way that increases adaptation and facilitates recovery to the extent possible. This model flies in the face of conventional medical concepts of homeostasis, but is entirely compatible with the tenets of existential positive psychology, as will be shown later. The second key tenet is that *this adaptation is influenced by individual and societal factors that aid in combating stressors*, and that prevent an individual exposed to stress from “breaking down,” or developing an overt disease. Antonovsky referred to these factors as general resistance resources (GRRs). GRRs may be biological (such as genetic predisposition or nutritional status), psychological (such as cognitive appraisals or emotional regulation), social (such as social support, religious or cultural practices), or material (such as the availability of financial resources) in nature. The third and perhaps most important facet, which provides the answer to the “salutogenic question,” is the concept of the sense of coherence (SOC). SOC is a multidimensional construct which encompasses *comprehensibility* (the ability to understand one’s problem or disease), *manageability* (the sense of having enough individual or external resources to cope with stressors and disease), and *meaningfulness*. This third dimension, which is based on the earlier work of Frankl (1954), refers to a sense that “life is worth living,” resulting in a motivation to adapt positively to one’s environment and find meaning even in adverse circumstances. Though SOC was initially developed as a concept applying to individuals, Antonovsky later extended it to communities, using it to explain the different responses of groups to a general stressor or crisis. A more extensive discussion of the finer details of this model can be found in the comprehensive review by Vinje et al. (2017).

### Salutogenic Approaches to Health, Particularly Mental Health

Salutogenic approaches to health, which flow directly from this model, include interventions aimed at increasing the SOC. These may take the form of developing shared adaptive models to understand an illness or stressor (comprehensibility), drawing upon or developing GRRs to address life’s demands (manageability), and developing a model of one’s life situation that includes meaning and a vision for the future (meaningfulness) (Bauer et al., 2020). A review of published research examining these approaches found that, despite reservations regarding methodological quality, they were effective in specific aspects such as improving symptoms of depression and anxiety, lowering infection rates with HIV, and reducing preventable mortality in certain medical conditions (Alvarez et al., 2020); the first of these effects is obviously of relevance to the current discussion. In a broader sense, researchers have also found validation of the salutogenic framework in the context of responses to severe forms of adversity, such as drought (Austin et al., 2020) and sexual abuse (Dube and Rishi, 2017). In the former paper, a higher SOC was associated with well-being in rural residents of a drought-affected area; in the latter, a number of GRRs were associated with positive outcomes, in terms of quality of life, in adult survivors of childhood sexual abuse. Constructs related to salutogenesis, such as measures of the SOC, have also been found to predict mental health outcomes in patients with chronic medical illnesses such as inflammatory bowel disease (Freitas et al., 2015) and cancer (Sales et al., 2014). Finally, researchers examining the effectiveness of a salutogenic approach to specific mental health problems have reported positive outcomes in child and adolescent emergency psychiatry settings (Johansson et al., 2018), and in psychosocial rehabilitation (Fekete et al., 2020).

The latter example is particularly illustrative and merits discussion at some length. Patients with chronic mental illness often show only partial responses to standard pharmacological therapies, and rehabilitation is often essential to improving their quality of life, functioning, and social inclusion (Farkas and Anthony, 2010). In a paper describing the implementation of a comprehensive psychosocial rehabilitation program in Norway, Fekete et al. (2020) have drawn on five key principles derived from the salutogenic framework: (1) health as a continuum, (2) focus on the “story” of an individual as whole rather than on a medical diagnosis, (3) GRRs, (4) the potentially adaptive aspect of tension or stress, and (5) the need for active adaptation to current circumstances. A focus on the holistic “story of a person” is of particular importance during the COVID-19 pandemic. Most popular and scientific descriptions of the psychological responses to COVID-19 pandemic have framed them as arising from an overwhelming stressor or sequence of stressors. In contrast to this, the “story of the person” approach emphasizes seeing individuals as “persons” rather than “patients” or “victims,” and attempts to see each person’s difficulties in the context of their broader life history and social context. Thus, for example, an individual experiencing “symptoms of anxiety” (see section “Methodological Issues”) because of financial losses due to a COVID-related lockdown has a quite different “life history” from another individual whose “symptoms of anxiety” represent an exacerbation of a pre-existing psychiatric illness. A purely descriptive or medical paradigm would treat both these persons almost identically, while a salutogenic approach would attempt to appreciate the factors peculiar to each individual, and to use this knowledge to draw upon appropriate GRRs. In this example, the second person might benefit from formal psychiatric treatment, while the first might actually perceive an offer of such treatment as dismissive, or as ignoring the particulars of his “story.” Such an approach is largely isomorphic with the concept of “person-centered medicine,” which emphasizes the individuality and contextual realities of a person and their adaptation to illness and adversity. In fact, the International College of Person-Centered Medicine (ICPCM) has acknowledged the central role of salutogenic concepts in promoting and preserving health and well-being during crises, such as wars, natural disasters and pandemics (Christodoulou et al., 2018).

It is also of significance that salutogenic factors and interventions based on them have been found to play an important role in health outcomes in the elderly, who are disproportionately affected both physically and psychologically by the COVID-19 pandemic (Grolli et al., 2021). For example, a sense of meaningfulness–one of the key components of the SOC–has been positively associated with mental health outcomes (Ninomiya et al., 2019) and adherence to healthy lifestyle practices (Stodle et al., 2019) in adults aged 65 and above. Similarly, interventions aimed at enhancing the SOC of elderly persons have been associated with improvements in self-reported mental health (Sundsli et al., 2014; Murayama et al., 2015). This suggests that the benefits of a salutogenic approach may extend across the entire life-span.

### Evidence for the Relevance of Salutogenic Factors During the COVID-19 Pandemic

There is already substantial evidence that various factors, which can easily be identified as GRRs using the above paradigm, are associated with increased resilience in the face of adversity during the COVID-19 crisis. Most of the GRRs reported in the literature thus far are of a psychological or social nature. For example, character strengths–such as the innate ability to withstand adversity or to maintain good interpersonal relationships–have been associated with better mental health and well-being during a COVID-19-related lockdown (Martinez-Marti et al., 2020). The use of coping strategies that focus more on problem-solving was associated with reduced psychological distress in nurses exposed to an increased workload during the pandemic (Lorente et al., 2020). Involvement in religious activities, even privately, was associated with reduced fear and worry in adults isolated during the pandemic (Lucchetti et al., 2020), and a similar relationship was found between religious faith and a sense of security in the face of COVID-19 (Kowalczyk et al., 2020). From a material perspective, access to unemployment insurance during the pandemic was associated with better mental health (Berkowitz and Basu, 2020). Conversely, it has been observed that individuals who were already living in adverse circumstances prior to the pandemic, and who had reduced access to several commonly available GRRs, were more likely to experience psychological distress during the pandemic (Banati et al., 2020).

Similarly, researchers assessing the role of the SOC in influencing mental health during the COVID-19 pandemic have found results that are broadly supportive of the salutogenic framework. In particular, a weak SOC was associated with an increased likelihood of “probable depression or anxiety disorder” across eight countries (Genereux et al., 2020); a similar result was obtained in samples of adults from Italy and Germany (Barni et al., 2020; Schafer et al., 2020). The authors of the latter paper explicitly recommended interventions aimed at enhancing SOC in vulnerable individuals. The validity of this approach was tested in a study of a recreational intervention in a small sample of women (*n* = 53), which found that over a period of 6 months, physical activity was associated with an increase in SOC; however, initial SOC was itself associated with a greater likelihood of adhering to the exercise regimen, suggesting a bi-directional relationship (Szovak et al., 2020).

These results, though provisional in nature, suggest that a salutogenic approach to the problem of psychological distress during the COVID-19 pandemic may be beneficial, particularly in individuals in the general population without a prior psychiatric diagnosis. The next question to be addressed is what such an approach would look like in the real world.

### Outlining a Salutogenic Approach to Psychological Distress During the COVID-19 Pandemic

Though the fine details of any intervention in this context would need to be tailored to cultural and logistic realities in a given setting, a broad outline of how such an approach might be constructed will now be outlined in broad terms. The first step in such an approach would be to assess the current level of psychological distress in a given individual, and particularly to perform a risk assessment regarding the potential for harm to self or others. This step would allow those requiring formal psychiatric management or hospital-based care to be “filtered out,” and would essentially be a sort of triage. However, once any acute risk in these individuals has been adequately managed, they may also benefit from the intervention model proposed here. The second step would be to attempt to understand the individual’s distress, not using formal diagnostic criteria, but in the context of his or her broader history. Relevant questions at this stage would include: How does the individual understand their current predicament? How have they reacted to adverse experiences or situations in the past? To what extent do they think they can handle the situation? What are the realities of their current situation before and after the impact of COVID-19? What vision did they have for their future prior to the pandemic, and how has this changed in the current context? From the answers to these questions, a sketch of the “story of the person” beyond his symptoms arises, even if some details may need to be filled in later. The third step would be to assess the individual’s current SOC, perhaps by using a valid structured instrument as described in the literature (Eriksson and Lindstrom, 2005), along with the enumeration of available GRRs, whether individual (How does the individual manage stress?), social (what are the significant relationships in the person’s life? What broader support systems or networks are available? What are the individual’s religious or spiritual beliefs and practices) or material (what resources are currently available in terms of food, clothing, shelter, or money? What sources of help are available and accessible?). The fourth step would involve developing a shared understanding of the current problems as an attempt to adapt, rather than as a mental disorder or illness. In the final step, potential interventions can be selected and tested in collaboration with the individuals. These may be tailored to address either of the three aspects of the SOC, depending on the individual’s needs. An outline of this process is provided in Table 1.

**TABLE 1:** Applying salutogenic principles in managing psychological distress in the context of COVID-19, in five steps.

Step	Brief description	Techniques and interventions used	Salutogenic principle(s) being applied
I	Triage	1. Screening for (a) acute risk of harm to self or others (b) diagnosable psychiatric disorder requiring formal treatment 2. Referral of individuals identified as (a) or (b) to “conventional” psychiatric care 3. Ensuring that such referred individuals return to the “salutogenic” intervention after acute management under (2)	Use of general resistance resources available within a conventional framework
II	History	Semi-structured interview assessing the following 1. Individual’s understanding or “explanatory model” of their current situation or predicament 2. Responses to adverse circumstances earlier in life 3. Sense of mastery over the current situation 4. Brief biographical sketch	Constructing a “story of the person” beyond symptoms or distress
III	Assessment	1. Enumeration of methods used to handle adversity in the past and present, especially those perceived as helpful 2. Enumeration of the individual’s perceived strengths and weaknesses 3. Enumeration of available general resistance resources–social, cultural or religious, material 4. If appropriate, structured assessment of the sense of coherence (SOC)	Identifying and preparing to mobilize existing general resistance resources (GRRs); baseline estimation of SOC
IV	Shared understanding	1. Explanation of the salutogenic model in common-sense or easily understandable terms 2. Reassurance that distress does not equate to mental disorder or illness and may be a “normal” response to an abnormal situation 3. Reframing the current situation, not just in terms of adversity, but in terms of an opportunity for growth and future positive mental health	Framing tension or stress as an opportunity for adaptation; preparing the individual for active adaptation rather than passive reception of medication or therapy
V	Salutogenic interventions	1. Select dimension(s) of the SOC which require specific attention for the individual–comprehensibility, manageability of meaningfulness. (a) Comprehensibility–develop a model of the current situation as a “challenge” and not just a “disaster” or “crisis”; Such a model must be open to revision and not “rigid.” Avoid either false hope or excessive pessimism (“realism”). Provide accurate information if the individual lacks access to this, or is misinformed. (b) Manageability–mobilize and optimize existing GRRs; develop and test potentially unexplored GRRs in collaboration with the individual; accept setbacks and non-linear improvement; focus on adaptation and not on specific symptoms. (c) Meaningfulness–focus the individual’s attention on the future “beyond” COVID-19, or at least “beyond” the immediate situation; develop short-term and long-term practical goals in terms of functioning and relationships. Call upon individually held “meanings” as well as shared wisdom from the prevailing culture or religious tradition.	Strengthening the SOC in a manner specific to the needs of the individual; optimal use of GRRs; construction of a “life story” that is not centered exclusively on COVID-19; active adaptation

In the context of the COVID-19 pandemic, however, particular emphasis must be laid on the fact that the individual is dealing not with a single stressor or event, but with a sequence of adverse events that often appear to be “endless” (Schippers, 2020). In this setting, the salutogenic approach outlined in this paper which is largely derived from prior literature in non-pandemic settings, may require certain adaptations. In particular, it would require a focus on the challenges of adapting to prolonged abnormal situations, which entail a certain and inevitable degree of suffering. How can individuals faced with this unprecedented situation understand their predicament, achieve a certain degree of mastery over it, and–more importantly–find meaning and opportunities for growth and character development over time? It is to answer these questions that we turn to the field of existential positive psychology (PP2.0), which, as will be shown, can be seen as the “missing link” in constructing a salutogenesis-based response to COVID-19.

## Existential Positive Psychology (PP2.0)

“Positive psychology” is an umbrella term which broadly refers to psychological research that is focused on the positive aspects of human experience, such as character strengths, positive emotions, resilience, and adaptive forms of coping or psychological defense. As such, it is entirely compatible with Antonovsky’s salutogenic model of health–in fact, one can state, without too much inaccuracy, that findings derived from positive psychology form an essential part of any salutogenic intervention aimed at improving mental health. More specificially, the parameters studied by positive psychology can all be considered psychological GRRs, and there is a strong correlation between resilience–a key concept in positive psychology–and the SOC that plays a central role in the salutogenic model (Lundman et al., 2010; Schrank et al., 2014).

While approaches based on the “first wave” of positive psychology (PP1.0) have shown promise in the field of mental health and substance abuse (Krentzman, 2013; Walsh et al., 2017), they also have certain limitations. From a methodological perspective, much research in this area is not based on a clear rationale or treatment goal, and the exact methods applied are often not described in sufficient detail to allow meaningful replication (Walsh et al., 2017). From a practical perspective at the community level, such concepts may be misused and misunderstood, and end up being reduced to simple catchphrases or slogans rather than translated into meaningful interventions (Cowen, 2001). A critique at a deeper level arises from the fact that “positive psychology” interventions, such as listing character or family strengths or enumerating one’s “blessings,” may not be appropriate in situations characterized by prolonged or severe adversity or a low likelihood of an eventual positive outcome.

It was as a result of this critique that the framework of existential positive psychology, sometimes referred to as PP2.0 (“the second wave of positive psychology”) was developed. Like Antonovsky’s salutogenic model, PP2.0 is based on the earlier work of Frankl (1954; 1966; 1972) on deriving meaning from suffering as part of the therapeutic process. PP2.0, like Antonovsky’s work, is fundamentally rooted in a critique of the medical model of mental health as incomplete or inadequate, particularly in the face of recent global social and economic changes and more specifically in the context of crises such as COVID-19 (Wong, 2020). PP2.0–like PP1.0–is not exclusively an approach related to health, as it also addresses questions such as organizational health and resilience (Mayer and Oosthuizen, 2020) and collaboration between cultures in a changing world (Barmeyer and Mayer, 2020); however, in this paper, it is the health-related aspects of this framework that will be discussed. A central aspect of PP2.0 is the acknowledgment that suffering and adversity, rather than being seen as “problems” to be “solved,” are an integral part of human existence–an insight that replicates the traditional wisdom of both Western and Eastern religious traditions (Fitzpatrick et al., 2016) as well as the salutogenic principle that tension or stress can be adaptive (Fekete et al., 2020). In other words, attempts to prevent or minimize suffering–as per the conventional medical model–may lead to a minimization or even a negation of the value of suffering, particularly in settings where such minimization is not feasible (Wong and Tomer, 2011; Wong, 2020). It is clear from the foregoing discussion that the COVID-19 pandemic and its sequelae represent one such setting. The therapeutic approach described by Wong (2020) in addressing this situation–which he has termed integrative meaning therapy (IMT)–is fundamentally spiritual rather than materialistic, in contrast to the medical model, and its two key tenets are self-transcendence and self-detachment. The integration of these principles into the salutogenic framework is described below.

### Integrating PP2.0 Into a Salutogenic Framework for Psychological Distress in the Context of the COVID-19 Pandemic

From the perspective of IMT, or of PP2.0 in general, humans are not purely material beings–rather, they are in a certain sense “hard-wired” for the transcendent and spiritual (Wong, 2020). This perspective is supported by empirical evidence suggesting that spirituality and religion are linked to positive outcomes in terms of physical and mental health and well-being (Braam and Koenig, 2019; Jaiswal et al., 2020; Moreira et al., 2020). Thus, attempting to address the problem of suffering during the COVID-19 pandemic in purely medical terms may be ineffective or even harmful. As an alternative to the medical model, and in keeping with the tenets of the salutogenic model, IMT suggests that self-transcendence–defined as seeking meaning in something higher than oneself, whether this is God, other people, or both–and self-detachment, defined as an attitude that discourages self-absorption and encourages mindfulness–can lead to an overall increase in what salutogenic theory would identify as the SOC (Wong, 2020). In opposition to the medical model, IMT suggests that growth and resilience are achieved by confronting and accepting the distress associated with the pandemic, rather than by trying to minimize it using pharmacotherapeutic or psychotherapeutic approaches. IMT expands the salutogenic approach proposed in section “Outlining a Salutogenic Approach to Psychological Distress During the COVID-19 Pandemic” by emphasizing three specific types of GRRs: psychological (confronting rather than avoiding fears, mindfulness, self-affirmations regarding the value or meaning of suffering), social (concern for others, altruism, strengthening existing relationships) and spiritual (belief in God, however this is understood by the individual, and therefore in a higher-order meaning or purpose beyond the current situation) (Wong, 2020). However, it would be erroneous to simply consider IMT as an “add-on” or “adjunct” to the salutogenic approach. Rather, it should be seen as a broader framework into which salutogenic principles can be placed, but which transcends it through an emphasis not just on meaning or coherence but on love–understood in the sense of willing the good of others (“mature love”) (Milivojevic and Ivezic, 2004; Levine, 2005) and not as a merely emotional or biological phenomenon (Stein and Vythilingum, 2009). A corollary of this is that a PP2.0/salutogenic intervention would, in this higher sense, be an “act of love” on the part of the therapist, helping individuals to grow and confront adversity, and not simply a “treatment” for a “medical condition” (Karasu, 1999; Wong, 2020).

To paraphrase Wong (2020), the goal of IMT is a life that is “purposeful, understandable, responsible, and enjoyable” (“PURE”) with the latter being understood in a sense beyond material or physical pleasure. Because of its fundamental orientation toward others, an IMT-based salutogenic framework has the potential to benefit not just individuals but entire communities faced with the COVID crisis (Kola et al., 2021). Such approaches have already shown some promise in individuals who have been exposed to severe forms of adversity (Kizilhan and Wenzel, 2020). Potential steps that would enable the integration of IMT into a salutogenic framework are described in Table 2.

**TABLE 2:** Integrating existential positive psychology (PP2.0) into a salutogenic approach to psychological distress during the COVID-19 pandemic.

Salutogenic principle	Corresponding PP2.0 principle(s) that may be integrated	Advantages
Health as a continuum	Limitations of the medical model; aspects of human suffering that cannot (and should not) be “medicalized”	Reduced stigma associated with “psychiatric” diagnosis and care; acceptance of distress as normal and understandable.
Focus on the story of the individual	Consideration of both “micro” (individual) and “macro” (socioeconomic and cultural) factors; aspects of self-transcendence (love of God and others) in the individual’s life	Facilitates the identification of general resistance resources and vulnerabilities; builds empathy and rapport with between individual and therapist
Adaptive nature of tension and stress	Reality and inevitability of suffering and adversity	Minimization of protest or complaint as unhelpful and not constructive; emphasis on positive coping; builds understandability
General resistance resources	Mindfulness Self-transcendence–includes religion and spirituality Love of others and altruism Focus on individual’s core values or virtues, and on their freedom to choose certain behaviors	Reduced distress and anxiety Improved well-being and reduced self-centeredness; builds meaningfulness Benefits family and community Builds manageability
Active adaptation to current circumstances	Well-being as balance between positive goals and negative forces or circumstances Confrontation, rather than avoidance, of adversity	Emphasis on process rather than strictly defined outcome; acceptance of setbacks Makes the individual an active participant in the therapeutic process; builds manageability

Once developed, such interventions would need to be tested rigorously to establish their advantages over conventional forms of supportive or psychological intervention. These studies would need to be conducted using sound methodology (Alvarez et al., 2020) and, ideally, to follow up subjects over a long period of time, as they are aimed not just at symptom relief but at fostering resilience and adaptation (Langeland and Vinje, 2017).

### Lessons for Psychiatry From PP2.0: Beyond the COVID-19 Pandemic

The principles derived from the integration of the salutogenic model and PP2.0 has implications that extend far beyond the COVID-19 pandemic. For years, theoreticians and researchers have warned their colleagues of the limitations of a purely medical approach to mental health, which can be summarized under five broad headings. First, there are certain aspects of human psychological suffering that may, by their very nature, not respond to conventional medical approaches (Aho, 2008; Clark, 2014). Second, the pervasiveness of the medical model and its emphasis on “symptom reduction” or “cure” has led to situations that border on the absurd, where even normal human sadness or unhappiness is “medicalized” (Mulder, 2008; Dura-Vila et al., 2011) and responses to social and political change are wrongly framed in terms of psychiatric diagnostic categories (Degerman, 2019). Third, the widespread acceptance of the medical model has led to the sidelining of other approaches to mental health promotion or case, particularly where psychological distress arises in the context of social adversity, deprivation or change (Haack and Kumbier, 2012). Fourth, the acceptance of the medical model as the “*status quo*” has led to a neglect of the adverse effects of conventional psychiatric treatment, which may range from the subtle to the life-threatening (Moncrieff, 2018). Fifth, the framing of all suffering or distress as a medical problem leads to a neglect of the ubiquity of such experiences, and their potential role in character growth (Clark, 2014; Sedler, 2016; Wong, 2020).

The approach outlined in this paper, based on salutogenesis and the second wave of positive psychology, can act as a corrective to some of these limitations. Such an approach could serve as the foundation for mental health approaches that are proactive, realistic, and grounded in the notion of positive mental health as a dynamic state of adaptation and growth, even in the face of adversity (Kola et al., 2021). Such approaches would allow clinicians and researchers to move beyond the “disease-centered” or “syndrome-centered” model that dominates psychological medicine, and to consider treatment approaches beyond pharmacotherapy or conventional forms of psychotherapy. Such an approach would be of particular use in (a) patients with psychological distress that does not fit into conventional diagnostic categories, (b) individuals and communities exposed to particularly severe or prolonged forms of trauma or adversity, (c) individuals from cultures in which the medical model is not predominant, (d) patients with chronic mental illness who have responded inadequately to standard medical treatments, and (e) patients with concurrent medical and psychiatric conditions, or even so-called “psychosomatic disorders,” who tend to “fall between the cracks” of a medically oriented healthcare system. In addition, such a model could also play a potential role in preventing the emergence of psychiatric “syndromes” in at-risk of vulnerable populations through enhancing adaptation and the SOC, and it would also be more respectful of cultural models–particularly those based in religion and spirituality–which cannot always be easily aligned with a medical-scientific perspective (Peteet, 2014).

The limitations of this approach must also be acknowledged. First, as the concepts of salutogenesis and PP2.0 are likely to be unfamiliar to many practitioners, a period of training and orientation would be required prior to the implementation of any intervention based on these principles, as otherwise the “interventions.” Second, the existing literature on salutogenic interventions is subject to significant methodological limitations in up to 75% of published reports (Alvarez et al., 2020); thus, any future interventions will need to be more rigorously designed and tested, to avoid the possibility of false-positive or false-negative results. Third, these interventions should be compared with more “conventional” approaches such as supportive counseling, to ensure that any benefits obtained are specific to a PP2.0 framework and not due to the non-specific effects of psychotherapies (Palpacuer et al., 2017). Protocols for such comparisons should be methodologically sound (Alvarez et al., 2020), and should ideally enroll subjects with “sub-syndromal” symptoms or distress rather than patients with syndromal psychiatric diagnoses, who would benefit more from traditional psychotherapy or pharmacotherapy. Fourth, the use of such an approach requires empathy and sensitivity, particularly when dealing with patients with high levels of distress or facing severe adversity (Langeland and Vinje, 2017), as otherwise exhortations to accept suffering or adapt to it may be misunderstood or rejected (Kizilhan and Wenzel, 2020; Wong, 2020). Fifth, the broad principles outlined in this article need to be adapted to the social, cultural and economic realities of specific situations, to ensure their acceptance by individuals and communities (Wendt and Gone, 2018; Furlong and Finnie, 2020).

## Conclusion

In principle, the integration of PP2.0 into a broader salutogenic framework for psychological intervention, particularly in the context of COVID-19, has many advantages over conventional approaches. The development of a formal therapeutic model or manual for such an intervention would require further inputs from a wide range of stakeholders, including (1) direct perspectives from persons affected by the pandemic across countries and cultures (Alipour et al., 2020; Mazumder et al., 2021); (2) discussions with healthcare workers handling mental health issues during the pandemic, to outline their perceptions of the “conventional” psychiatric approach and the limitations thereof (Bommersbach et al., 2021); (3) expert opinions from specialists already involved in interventions based on a salutogenic, PP2.0, or person-centered approach (Christodoulou et al., 2018; Wong, 2020); (4) inputs from local community and spiritual leaders, to identify those aspects of meaningfulness and coherence that are culturally relevant and could foster resilience (Thompkins et al., 2020); (5) the perspectives of social science experts and policy makers on which systemic interventions could foster a SOC and strengthen resistance resources (Christodoulou et al., 2018; Alvarez et al., 2020); (6) specific advice from experts in information technology, in order to devise optimal strategies on adapting salutogenic-PP2.0 principles to interventions delivered via mobile or social media platforms (Goransson et al., 2020), and (7) careful planning of intervention trials in collaboration with experts in biostatistics and research methodology, to avoid the methodological flaws that affected earlier studies in this field (Alvarez et al., 2020). It is hoped that the preliminary steps outlined in this paper are of use not only to researchers, but to clinicians and those involved in formulating policies to help those most affected by the COVID-19 pandemic. The lessons learned in doing so may lead to the development of more a secure framework for general psychiatric practice, particularly in those patients whose suffering does not fit into a neatly defined diagnostic category. As a recent review has noted, global mental health needs to be “reimagined” in the aftermath of the COVID-19 pandemic, particularly in low- and middle-income countries, by moving away from “a narrow biomedical approach” to a more integrative, community-based model informed by psychological, social and spiritual factors (Kola et al., 2021). The potential synthesis of the salutogenic and PP2.0 models outlined in this paper offers one avenue by which such a “reimagination” could take place, and could supplement rather than supplant the existing medical-psychological paradigm. An emphasis on salutogenesis could empower patients and caregivers to take a more active role in managing their symptoms and in adapting to the varying circumstances of their lives, and could strengthen community resources and support networks by minimizing reliance on healthcare professionals alone.

## Author Contributions

RR developed the concept for this manuscript, carried out the literature search, critically analyzed the relevant literature, wrote the manuscript, and proofread it. This manuscript represents the author’s original work and has not been submitted for publication elsewhere.

## Conflict of Interest

The author declares that the research was conducted in the absence of any commercial or financial relationships that could be construed as a potential conflict of interest.
